# A RARE CASE OF PLEURAL LYMPHOMA

**DOI:** 10.4103/0970-2113.45283

**Published:** 2008

**Authors:** Sumitra Basuthakur, Anirban Sarkar, Sushanta Burman, Rajesh Dandale

**Affiliations:** 1 Professor, Dept.of TB & Respiratory Medicine, Medical College, 88, College Street, Kolkata; 2 Assistant Professor, Dept.of TB & Respiratory Medicine, Medical College, 88, College Street, Kolkata; 3,4 Postgraduate Trainee, Dept.of TB & Respiratory Medicine, Medical College, 88, College Street, Kolkata

**Keywords:** Primary Pleural Lymphoma, Non-Hodgkin Lymphoma, CT guided FNAC

## Abstract

We present a case report of a 20 years old male who had low grade fever, weight loss of about 10 kg and left-sided chest pain increasing in intensity over a year. Clinically, it mimicked left sided pleural effusion with a tender, soft, parietal swelling in left in-fraaxillary area. Chest x-ray and Computerized Tomography-scan of thorax showed pleura based mass in left hemi thorax. Computerized Tomography guided Fine Needle Aspiration Cytology confirmed the diagnosis of non Hodgkin Lymphoma, diffuse large B cell type, high-grade.

## INTRODUCTION

Primary Pleural Lymphoma is a very rare occurrence. Most of the documented cases have either presented with or later on developed pleural effusion in the course of the disease^1^. Malignant Lymphoma of the pleura has been mostly associated with chronic pleural inflammation. Usually a history of chronic longstanding tubercular pyothorax or pulmonary tuberculosis treated with artificial pneumothorax precedes development of pleural lymphoma^2^. The case under discussion is worth reporting because it has no history of pleuro-pulmonary disease, no associated pleural effusion and no evidence of lymphoma at any other site.

## CASE REPORT

A 20-year male, smoker with a smoking history of 10 cigarettes/day for 4 years, nondiabetic, normotensive, building construction worker, was admitted in the department of Respiratory Medicine, Medical College & Hospitals, Kolkata, with the complaints of left sided chest pain and low grade fever for one year. Fever was 99°–101° F, without chill or rigor, came mostly in the evening and subsided with perspiration within 3–4 hours without medication. The pain was continuous dull aching in character, gradually increasing in intensity to the extent of disturbing sleep at the time of admission. There was no cough, shortness of breath, wheeze or hemoptysis. He lost 10kg. of body weight over 1 year.

On general examination, he preferred left lateral decubitus. There was a soft tissue swelling in the left infraaxillary region. It was firm, very tender, about 2 cm in diameter, non fluctuant and with restricted mobility. Examination of Respiratory System revealed abnormal shape of the chest due to the swelling in the left infraaxillary area. Movement of left side was restricted. Trachea was shifted to right. Apex beat was not palpable. Dull percussion note and diminished vesicular breath sound was noted along left midclavicular line 4th space downwards and along left midaxillary line 5th space downwards. Right side of chest was normal on examination. Examination of other systems revealed no abnormality.

Blood examination (hemogram, liver function tests, urea, creatinine, sodium and potassium) and induced sputum for malignant cells and AFB smear examination showed no abnormality. Serial chest x-rays up to 5.2.07. showed increasing homogenous opacity occupying left mid and lower zone with obliteration of left costophrenic angle without any mediastinal shift. Percutaneous thoracentesis was unsuccessful. CT scan of thorax revealed pleural based soft tissue mass with irregular margin without any effusion in left hemithorax. CT guided FNAC and a tru cut biopsy from the mass reported non Hodgkin Lymphoma high grade, diffuse large B cell type.

USG of abdomen did not show any organomegaly, lymphadenopathy or ascites. Patient could not afford a contrast enhanced CT scan of neck and abdomen. His bone marrow examination showed normal report. His serum LDH level was 1904U/ml.

He was treated with Cyclophosphamide, Vincristine, Adriamycin and Prednisolone as per oncologist's advice. Unfortunately he expired of febrile neutropenia after 2nd cycle of chemotherapy following a good clinical response after 1st cycle of chemotherapy.

## DISCUSSION

Malignant lymphoma arising from lung and pleura is very rare. It accounts for about 0.3%of all non Hodgkin Lymphomas. Most of the reports have been found to be associated with longstanding chronic Tubercular Pyothorax or Pulmonary Tuberculosis treated with therapeutic Artificial Pneumothorax. Most of the reported case studies have suggested that, chronic inflammation of the pleura poses a significantly increased risk of developing pleura based lymphoma.^2^

Primary Pleural Lymphoma is extremely rare. In contrast, pleural involvement secondary to systemic lymphoma is relatively common. Approximately 16% of non Hodgkin Lymphomas either present with or subsequently develop pleural involvement, which is unilateral or bilateral. In case of unilateral involvement it is more common on the left side.^3^

The patients are symptomatic, mostly presenting with dyspnea, cough or chest pain. Pleural effusion commonly accompanies pleural lymphoma, which carries a poor prognosis.^4^

The radiological abnormalities are; a pleura based mass, pleural thickening, pleural effusion, mediastinal lymph nodes, bilateral pulmonary nodules etc. left pleural involvement is more common than the right or bilateral.^5^

Pleural biopsy and pleural fluid cytological examination are the early steps in the essential diagnostic workup of pleural-based mass.^6^

We presented here an extremely rare case of pleural lymphoma. It represented primary pleural lymphoma, as there was no evidence of any other site of involvement at the time of diagnosis. Also it was not associated with pleural effusion, which itself is another rare form of association of pleura and lymphoma. Neither there was any history of chronic pyothorax, nor any other form of chronic pleural inflammation, as has been described in the literature. However, a contrast enhanced CT scan of neck and abdomen are helpful to firmly establish the primary nature.

**Fig 1:**
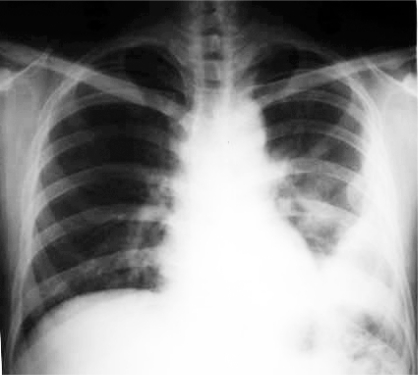
X-ray chest showing left pleural based mass.

**Fig 2:**
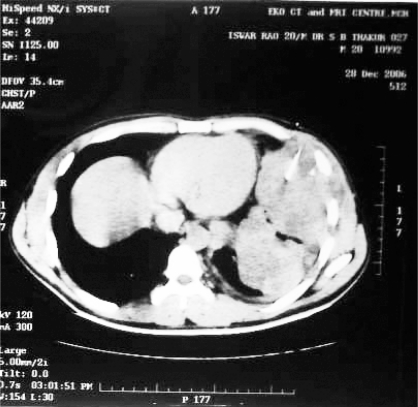
Computed tomography of the same lesion. A CT guided FNAC was taken from the mass

**Fig 3:**
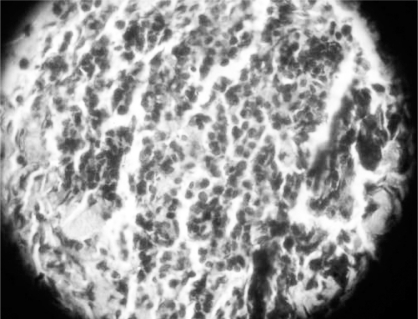
High power microscopy showing histopathological picture of non-hodgkin’s lymphoma.

For the last decade, cyclophosphamide, doxorubicin, vincristine, and prednisone (CHOP) has been the best available standard of care for non-Hodgkin Lymphoma (NHL), based on equivalent therapeutic results with other multiagent chemotherapy accompanied by lower costs and lesser toxicity. However, only 40—45% of these patients are cured with CHOP. There is now accumulating evidence that the clinical behavior of certain NHL can be profiled by the expression of certain molecular markers, which will undoubtedly play a role in the development of new prognostic models that may refine our ability to identify poor-risk patients7. Regardless, there is still significant opportunity for improving survival in large cell lymphomas.
